# Phylogeny and morphology of *Lasiodiplodia* species associated with *Magnolia* forest plants

**DOI:** 10.1038/s41598-019-50804-x

**Published:** 2019-10-04

**Authors:** Nimali I. de Silva, Alan J. L. Phillips, Jian-Kui Liu, Saisamorn Lumyong, Kevin D. Hyde

**Affiliations:** 1 Department of Biology, Faculty of Science, Chiang Mai University, Chiang Mai, 50200 Thailand; 2 Biodiversity and Ethnobiology, Department of Biology, Faculty of Science, Chiang Mai University, Chiang Mai, 50200 Thailand; 3 Center of Excellence in Microbial Diversity and Sustainable Utilization, Faculty of Science, Chiang Mai University, Chiang Mai, 50200 Thailand; 4 Key Laboratory for Plant Biodiversity and Biogeography of East Asia (KLPB), Kunming Institute of Botany, Chinese Academy of Science, Kunming, 650201 P.R. China; 5 Center of Excellence in Fungal Research, Mae Fah Luang University, Chiang Rai, 57100 Thailand; 6 World Agro Forestry Centre, East and Central Asia, 132 Lanhei Road, Kunming, 650201 P.R. China; 7 Universidade de Lisboa, Faculdade de Ciências, Biosystems and Integrative Sciences Institute (BioISI), Campo Grande, 1749- 016 Lisbon, Portugal; 8 School of Life Science and Technology, University of Electronic Science and Technology of China, Chengdu, 611731 P.R. China; 9 Academy of Science, the Royal Society of Thailand, Bangkok, 10300 Thailand

**Keywords:** Fungal biology, Fungal ecology

## Abstract

Two new species of *Lasiodiplodia* (*Lasiodiplodia endophytica* and *Lasiodiplodia magnoliae*) are described and illustrated from *Magnolia* forests in Yunnan, China. Endophytic and saprobic *Lasiodiplodia pseudotheobromae* and endophytic *L*. *thailandica* are new records from this host. The internal transcribed spacers (ITS), part of the translation elongation factor-1α (*tef1*) and partial β-tubulin (*tub2*) sequence data were analyzed to investigate the phylogenetic relationships of the new species with other *Lasiodiplodia* species. *Lasiodiplodia magnoliae* is phylogenetically sister to *L*. *mahajangana* and *L*. *pandanicola* but morphologically distinct from *L*. *mahajangana* in having larger conidia. *Lasiodiplodia endophytica* is most closely related to *L*. *iraniensis* and *L*. *thailandica* and the three species can be distinguished from one another by 2 base pair differences in ITS and three or four base pair differences in *tef1*. The new collections suggest that *Magnolia* forest plants are good hosts for *Lasiodiplodia* species with endophytic and saprobic life-styles.

## Introduction

*Magnolia* species are widely distributed in temperate and tropical South East and East Asia. The wood is used extensively for the interior finish of houses and for door panels, e.g. *Magnolia champaca*, while the bark of *Magnolia officinalis* and other species is used in China as a valuable drug^1^. Many species of *Magnolia* and their hybrids are cultivated in gardens, grown as temple trees, and the flowers are used for decoration^1^.

During an investigation of Ascomycetes in sub-tropical regions of Yunnan, China we collected samples from *Magnolia* trees. Several isolates were of saprobic asexual fungi with hyaline and brown conidia bearing longitudinal striations and conspicuous conidiomatal paraphyses. These initial morphological observations suggested that the isolates are *Lasiodiplodia* species. *Lasiodiplodia* Ellis & Everh. is a genus in the family *Botryosphaeriaceae* (Botryosphaeriales, Dothideomycetes, Ascomycota)^2–4^ and typified by *L*. *theobromae* (Pat.) Griffon & Maubl.^3,5–7^. *Botryosphaeriaceae* forms a monophyletic lineage with 22 genera that are defined according to morphology of ascospores and conidia, and phylogenetic relationships^4^. The family is characterized by large, ovoid to oblong, usually hyaline, aseptate ascospores and hyaline or pigmented, aseptate, one or rarely multi-septate, thick walled conidia usually with longitudinal striations^4,7^. Considering asexual characters i.e. especially conidia characters, *Lasiodiplodia* species differ from other closely related genera in the *Botryosphaeriaceae* by the presence of pycnidial paraphyses and longitudinal striations on mature conidia^3^ while morphology (especially dimensions) of conidia and paraphyses is used for species delimitation^7,8^. Some genera of *Botryosphaeriaceae* show similar morphological affinities to *Lasiodiplodia* and some morphological characters can be used to distinguish these taxa from *Lasiodiplodia*^7^. As an example, *Barriopsis* species have ovoid conidia with striations even clearly visible in hyaline immature stage as well as pigmented mature stage^7,9^. The striated, pigmented, mature, ovoid conidia suggest close resemblances to *Lasiodiplodia* but the early development of striations in hyaline immature stage is a unique character for *Barriopsis*^7,9^. Another example is the genus *Neodeightonia* that shows close affinity to *Lasiodiplodia* in having striations pigmented mature conidia and can be differentiate from *Lasiodiplodia* by the absence of conidiomatal paraphyses^7,9^. However, on account of morphological variability within species, morphology alone is not reliable for distinguishing different *Lasiodiplodia* species. Phillips *et al*.^4^ suggest that combined LSU and ITS provide reliable resolution for phylogeny of Botryosphaeriales. However, protein coding genes such as *tef1* and *tub2* in addition to LSU and ITS provide greater support for species and genera level delimitation in order Botryosphaeriales^4^. In previous studies, phylogenetic analyses were solely based on ITS nucleotide sequences^3^ to identify *Lasiodiplodia* species. Inclusion of *tef1* sequences gives better resolution of phylogenetic relationships among species^3,6^. The recent multi locus phylogenetic approaches with ITS, *tef1* and *tub2* nucleotide sequence data has advanced the recognition of numerous *Lasiodiplodia* species with high phylogenetic support^3,4,10,11^. In that respect, sequence data of the internal transcribed spacers (ITS), partial translation elongation factor-1α (*tef1*) and partial β-tubulin (*tub2*) are now relied on for resolution of species in *Lasiodiplodia*^11^. There are 55 epithets of *Lasiodiplodia* recorded in Index Fungorum (March 2019) and 43 species names in MycoBank (March 2019). However, cultures and DNA sequence data are available for only 35 species (March 2019)^10–12^.

Species of *Lasiodiplodia* are cosmopolitan in tropical and subtropical regions and occur on a wide range of monocotyledonous, dicotyledonous and gymnosperm hosts^2,3,6,8,13^. They exhibit diverse life-styles as endophytes, inhabiting different asymptomatic plant tissues^8,14,15^, pathogens that cause diseases in various plant hosts^3,16^ and saprobes that are commonly found on dead woody plant tissues^3,17^.

To the best of our knowledge there have been no studies on the *Lasiodiplodia* species associated with *Magnolia* species in Yunnan Province, China. The aim of this study was to characterize *Lasiodiplodia* isolates in terms of morphology and phylogeny based on ITS, *tef1* and *tub2* sequence data.

## Results

### Phylogenetic analyses

**Figure 1:**
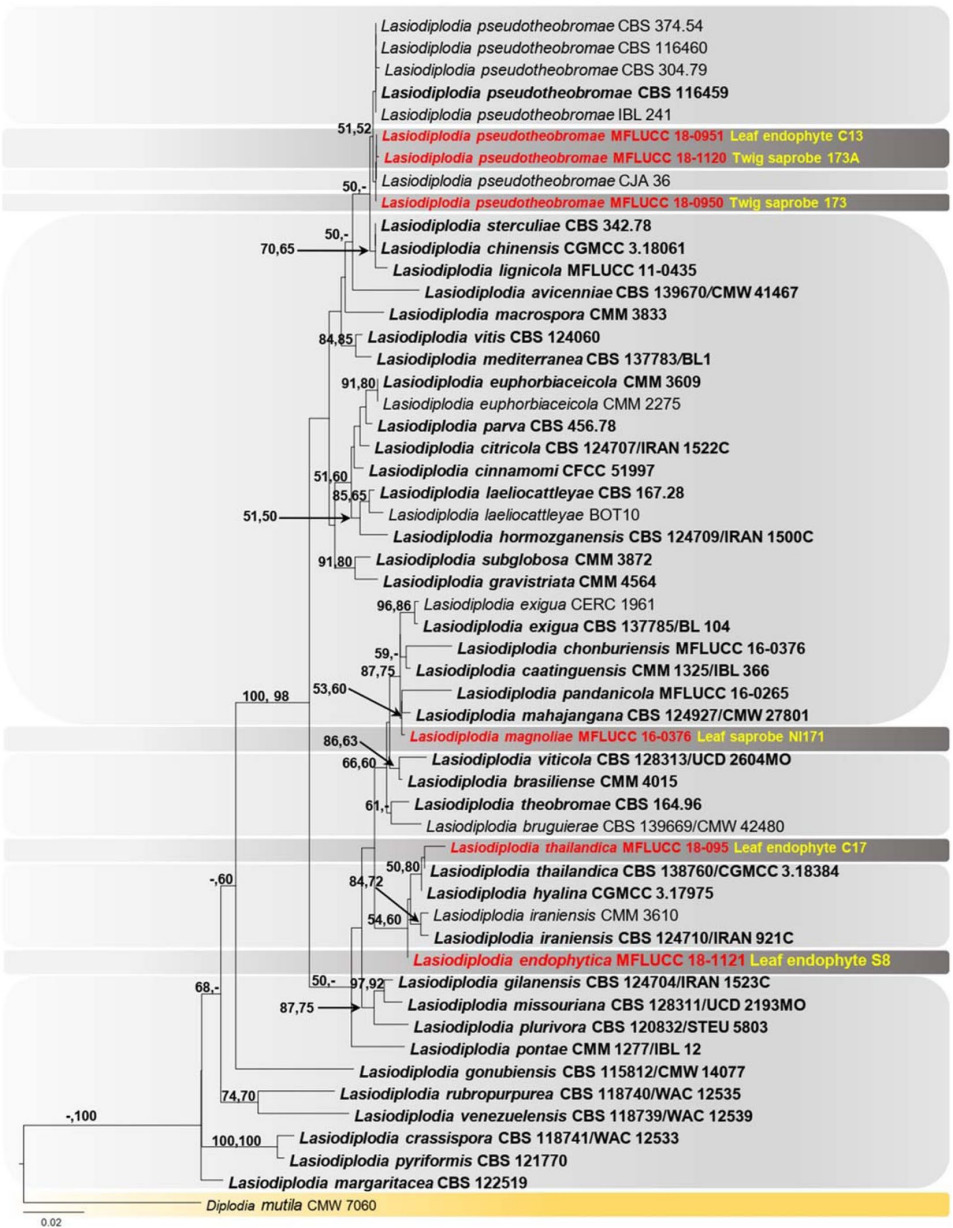
Maximum likelihood tree resulting from analysis of the combined ITS, *tef1* and *tub2* sequence data alignment. Bootstrap values for maximum likelihood (ML, first set) greater than 50, and maximum parsimony (MP, second set) greater than 50 are indicated at the nodes. The tree is rooted with *Diplodia mutila* (CMW 7060). The newly isolated strains are indicated in red bold and ex-type strains are indicated in black bold.

The combined dataset of ITS, *tef1* and *tub2* consisted of 54 taxa of *Lasiodiplodia*, with *Diplodia mutila* (CMW 7060) as the out group taxon and comprised 1267 characters including gaps after alignment. Of these, 1011 were conserved and 123 variable characters were parsimony uninformative. Maximum parsimony analysis of the remaining 133 parsimony informative characters resulted in 1000 equally parsimonious trees of 535 steps with CI = 0.632, RI = 0.798, RC = 0.504 and HI = 0.368. The maximum likelihood analysis resulted in a tree with largely the same topology as the maximum parsimony tree. The RAxML analysis yielded a best scoring tree with the final ML optimization likelihood value of - 4851.693940 (ln). Estimated base frequencies were as follows; A = 0.209292, C = 0.303982, G = 0.256083, T = 0.230643; substitution rates AC = 1.189236, AG = 3.165454, AT = 1.301265, CG = 1.047358, CT = 4.430504, GT = 1.000000; gamma distribution shape parameter α = 0.612671 (Fig. 1).

Four of the isolates from *Magnolia* clustered with known species, three with *Lasiodiplodia pseudotheobromae* and one with *L*. *thailandica*. The remaining two isolates formed distinct lineages representing two new species. *Lasiodiplodia magnoliae* was clustered separately and sister to *L*. *mahajangala* and *L*. *pandanicola* and *L*. *endophytica* formed a separate lineage and sister to *L*. *iraniensis* and *L*. *thailandica*.

### Taxonomy

**Description of** ***Lasiodiplodia magnoliae*** **N**.**I**. **de Silva**, **A**.**J**.**L**. **Phillips & K**.**D**. **Hyde**, **sp**. **nov**.

**Figure 2:**
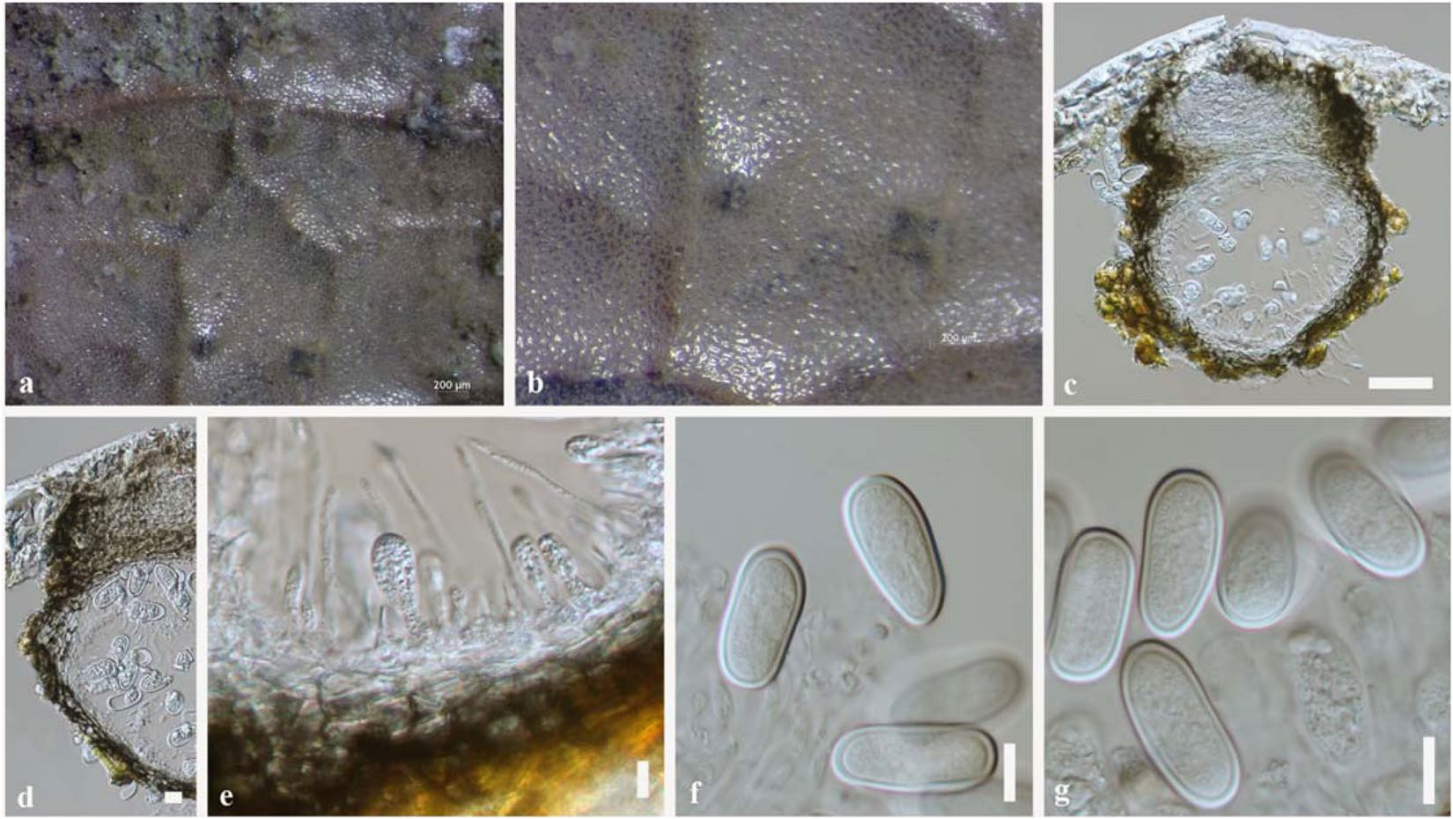
*Lasiodiplodia magnoliae* (MFLU 18-1030, holotype). (**a**,**b)** Appearance of conidiomata on dead leaf of *Magnolia candolii*. (**c)** Vertical section through conidioma. (**d)** Peridium. (**e)** Conidiogenous cells and paraphyses. (**f**,**g)** Conidia. Scale bars: c = 50 μm, d = 10 μm, e = 5 μm f, g = 10 μm.

Index Fungorum number: IF556217, Faces of Fungi number: FoF 05797 Fig. 2

Etymology – the epithet “*magnoliae*” refers to the host plant from which the taxon was collected.

Holotype: MFLU 18-1030

*Saprobic* on dead leaves attached to the tree of *Magnolia candolii* **Asexual morph:** *Conidiomata* 180–200 µm diam., 200–250 µm high, globose to subglobose, dark brown to black, scattered, solitary, immersed and uniloculate without a conspicuous ostiole. *Paraphyses* up to 60–70 μm long, 2–4 μm wide, hyaline, cylindrical, septate and rounded at apex. *Conidiophores* absent. *Conidiogenous cells* 2–5 µm diam., hyaline, discrete, smooth and cylindrical. *Conidia* (24–)25–27(–30) × 11–15 μm, hyaline, aseptate, ellipsoid to ovoid, with granular content, both ends broadly rounded, wall <2 μm thick.

Colonies on PDA reaching 30 mm diameter after 1 week at 20–25 °C, colonies medium sparse, circular, flat, surface slightly rough with edge entire, margin well-defined, cottony to fairly fluffy with sparse aspects, colony from above grayish-green to black with fluffy appearance reverse black.

#### Material examined

China, Yunnan Province, Xishuangbanna, dead leaves attached to the tree of *Magnolia candolii* (Magnoliaceae), 26 April 2017, N. I. de Silva, NI171 (Holotype MFLU 18-1030; Isotype HKAS100663), ex-type living cultures MFLUCC 18-0948, KUMCC 17-0198.

#### Notes

The combined ITS, *tef1* and *tub2* phylogeny showed that *Lasiodiplodia magnoliae* (MFLUCC 18-0948) clades sister to *L*. *mahajangana* and *L*. *pandanicola* with low support (53% ML, 60% MP) (Fig. 1). DNA sequence comparisons of ITS and *tef1* among *L*. *magnoliae*, *L*. *mahajangana* and *L*. *pandanicola* are given in Table 1. Comparison of total length of 445 bases of ITS sequences revealed one base pair difference among *L*. *magnoliae*, *L*. *mahajangana* and *L*. *pandanicola*. Comparison of total length of 450 bases of *tef*1 sequences revealed an insertion of eight bases in *Lasiodiplodia magnoliae* when compared to *L*. *mahajangana* and *L*. *pandanicola* (Table 1). *Lasiodiplodia magnoliae* has longer conidia (24–30 μm) than *L*. *mahajangana* (14–24 μm)^18^. *Lasiodiplodia pandanicola* has overlapping range of conidial dimensions (14–38 μm)^10^ with *L*. *magnoliae*. *Lasiodiplodia magnoliae* has longer paraphyses (60–70 μm) than *L*. *mahajangana* (27–66 μm)^17^. These three species were found on different host species and with different geographic distributions. *Lasiodiplodia magnoliae* was isolated from *Magnolia candolii* in Yunnan, China. *Lasiodiplodia mahajangana* was isolated from *Terminalia catappa* in Madagascar^18^. *Lasiodiplodia pandanicola* was isolated from dead leaves of *Pandanus* in Thailand^10^.

**Table 1:** Nucleotide differences between *Lasiodiplodia magnoliae* (MFLUCC 18-0948) and ex-type isolates of *L*. *mahajangana* and *L*. *pandanicola*.

Locus	ITS	*tef1- α*							
Nucleotide position	12	15	16	17	18	19	20	21	22
*L*. *magnoliae* MFLUCC 18-0948 (NI171)	G	C	G	G	C	G	C	T	G
*L*. *mahajangana*	A	—	—	—	—	—	—	—	—
*L*. *pandanicola*	A	—	—	—	—	—	—	—	—

**Figure 3:**
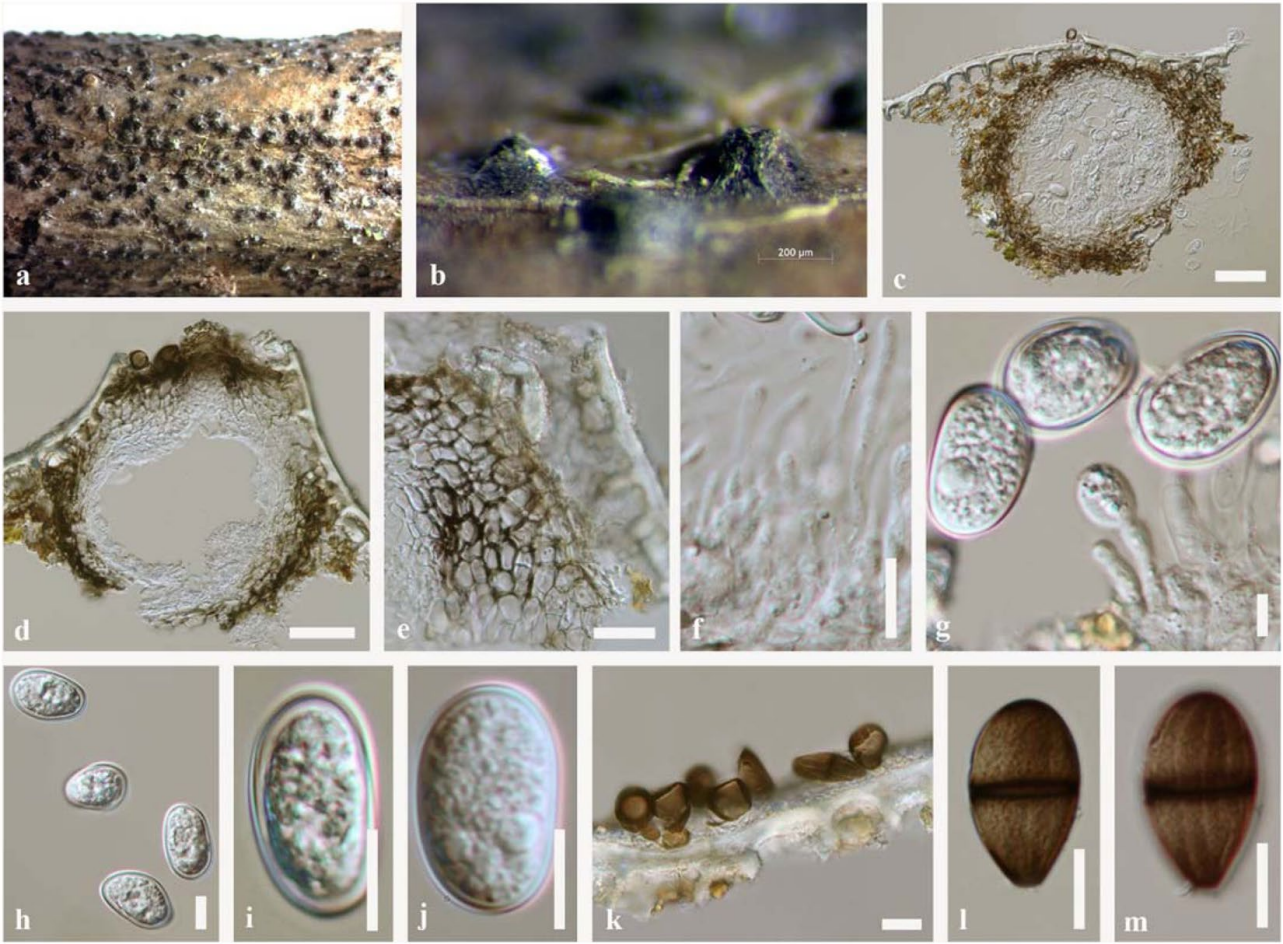
*Lasiodiplodia pseudotheobromae* (MFLUCC 18-1120, MFLUCC 18-0950). (**a**,**b)** Appearance of conidiomata on twig of *Magnolia* species. **(c**,**d)** Vertical sections through conidiomata. (**e)** Peridium. (**f)** Paraphyses (**g)** Conidiogenous cells. (**h–j)** Hyaline conidia. (**k)** Brown conidia on the surface of host. (**l**,**m)** Brown conidia. Scale bars: c, d = 50 μm, e, f = 20 μm, g = 5 μm, h**–**m = 10 μm.

**Figure 4:**
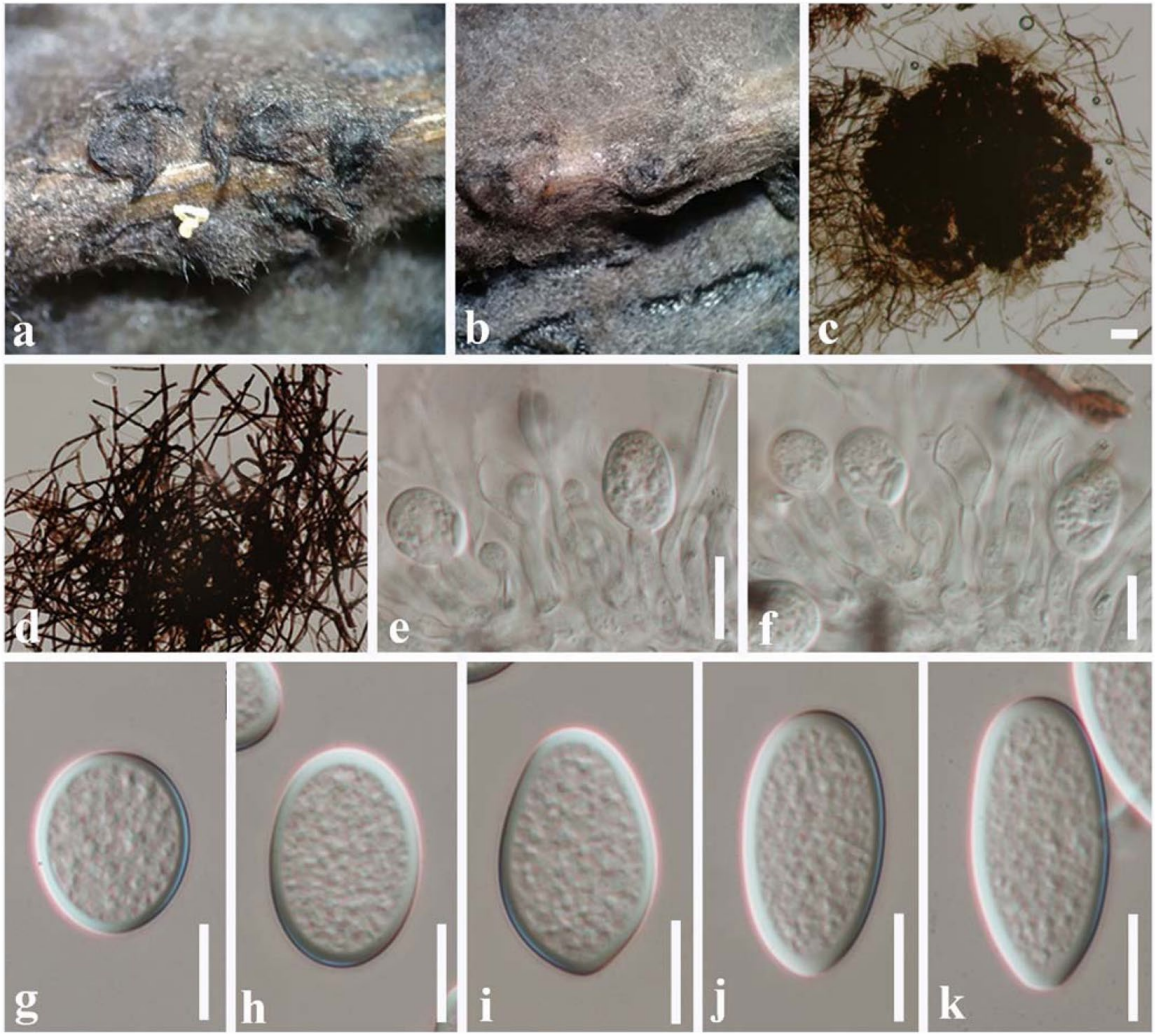
*Lasiodiplodia pseudotheobromae* (MFLUCC 18-0951). (**a**,**b)** Conidiomata on bamboo sticks in PDA culture plate. (**c)** Squash mount of conidiomata (**d)** Mycelium (**e**,**f**) Conideogeous cell (**g–k**) Conidia. Scale bars: c = 50 μm, e, f = 10 μm, g**–**k = 10 μm.

***Lasiodiplodia pseudotheobromae*** Alves & Crous, Fungal Diversity 28: 8 (2007). Figs 3 and 4

See Alves *et al*. (2007).

#### Material examined

China, Yunnan Province, Xishuangbanna, dead twigs (attached to the tree) of *Magnolia candolii* (Magnoliaceae), 26 April 2017, N. I. de Silva, NI173 (MFLU 18-1032, HKAS100665), living culture, MFLUCC 18-1120, KUMCC 17-0200; NI173A (HKAS100666), living culture MFLUCC 18-0950, KUMCC 17-0201; *Ibid*., fresh leaves of *Magnolia candolii* (Magnoliaceae), 26 April 2017, N. I. de Silva, C13; living culture, MFLUCC 18-0951, KUMCC 17-0218.

#### Notes

According to the combined ITS, *tef1* and *tub2* phylogeny, two isolates NI173 and NI173A from *M*. *candolii* twigs clustered with *Lasiodiplodia pseudotheobromae* with low support (51% ML, 52% MP) (Fig. 1). Conidia of these two isolates were hyaline, (20–26 × 10–14 μm) and brown (19–25 × 12–15 μm) and thus are smaller than in the ex-type isolate (27.5–28.5 × 15.5–16.5 μm)^6^. One endophytic strain (C13) from the same *M*. *candolii* plant was phylogenetically closely related to *L*. *pseudotheobromae* and clustered with two saprobic strains. Conidial dimensions of the endophytic isolate (26–31 × 10–12 μm) overlap with those of the ex-type isolate. The type of *L*. *pseudotheobromae* was isolated from *Gmelina arborea* in Costa Rica and has also been recorded from *Citrus* sp., *Coffea* sp.^3^, *Pteridium aquilinum*^19^, and *Plukenetia volubilis*^20^. Here we record endophytic and saprobic *L*. *pseudotheobromae* for the first time on *Magnolia candolii* in Yunnan, China.

***Lasiodiplodia thailandica*** T. Trakunyingcharoen, L. Lombard & Crous, *Persoonia* 34: 95 (2015) Fig. 5

**Figure 5:**
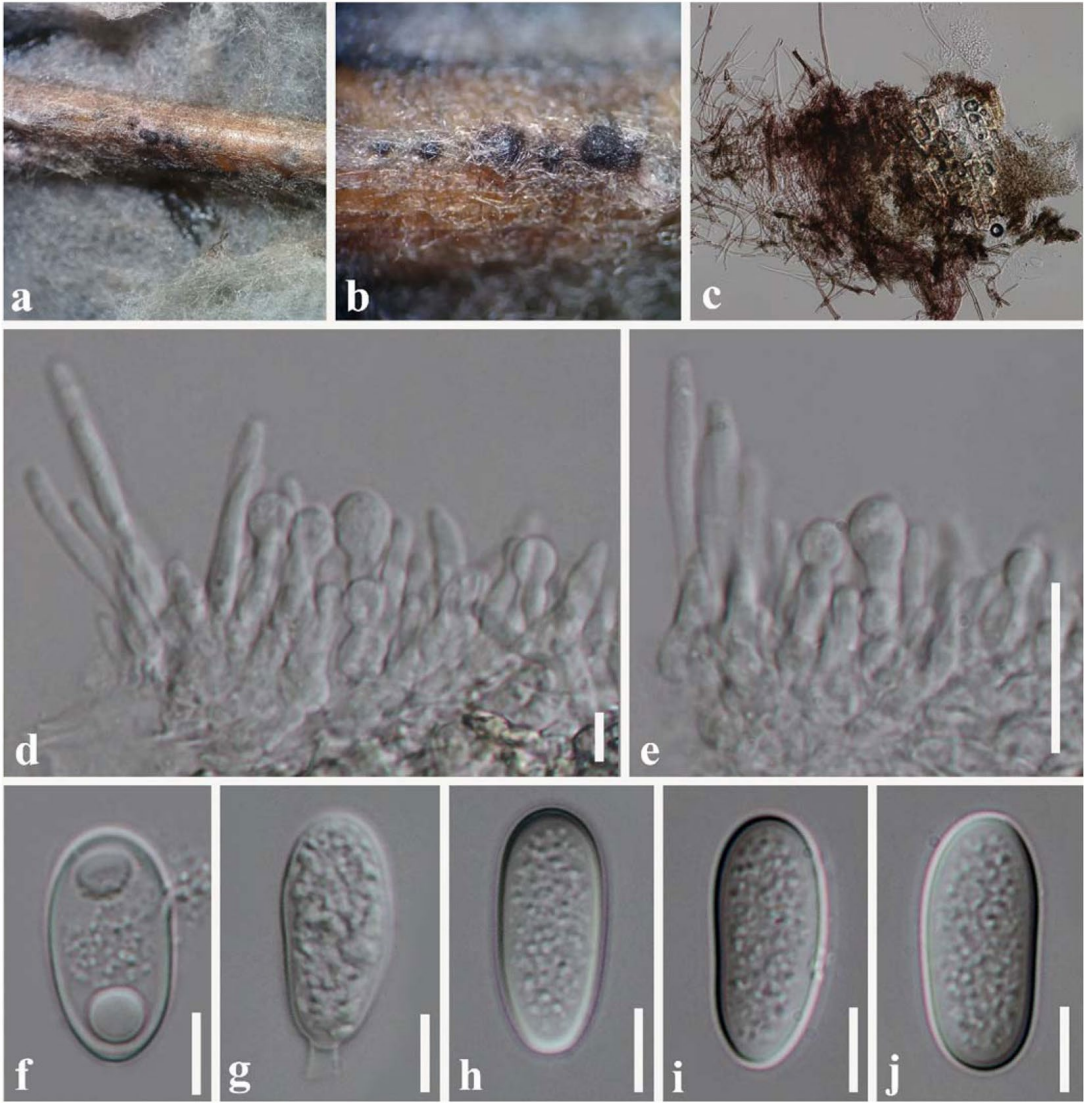
*Lasiodiplodia thailandica* (MFLUCC 18-0952). (**a**,**b)** Conidiomata on bamboo sticks in PDA culture plate. (**c)** Squash mount of conidiomata (**d)** Conideogeous cell (**e)** Paraphyses (**f–j**) Conidia. Scale bars: d = 5 μm, e = 20 μm, f**–**j = 10 μm.

See Trakunyingcharoen *et al*. (2015).

#### Material examined

China, Yunnan Province, Xishuangbanna, fresh leaves of *Magnolia candolii* (Magnoliaceae), 26 April 2017, N. I. de Silva, C17; living culture, MFLUCC 18-0952, KUMCC 17-0222.

#### Notes

Phylogenetically, the new isolate clustered with the ex-type isolate of *Lasiodiplodia thailandica* (CBS 138760) based on combined ITS, *tef1* and *tub2* sequence data. However, the new isolate has larger conidia (28–29 × 11–13 μm) than the ex-type of *L*. *thailandica* (20–26 × 12–16 μm)^21^. *Lasiodiplodia thailandica* was first described from symptomless twigs of *Mangifera indica* in Chiang Mai province, Thailand^21^ and also has been recorded from a petiole of *Phyllanthus acidus* in Thailand^20^, from cankered branch of *Podocarpus macrophyllus* in China^19^ and from cankered branch of *Albizia chinensis* in China^19^.

***Lasiodiplodia endophytica*** N.I. de Silva, A.J.L. Phillips & K.D. Hyde, sp. nov.

**Figure 6:**
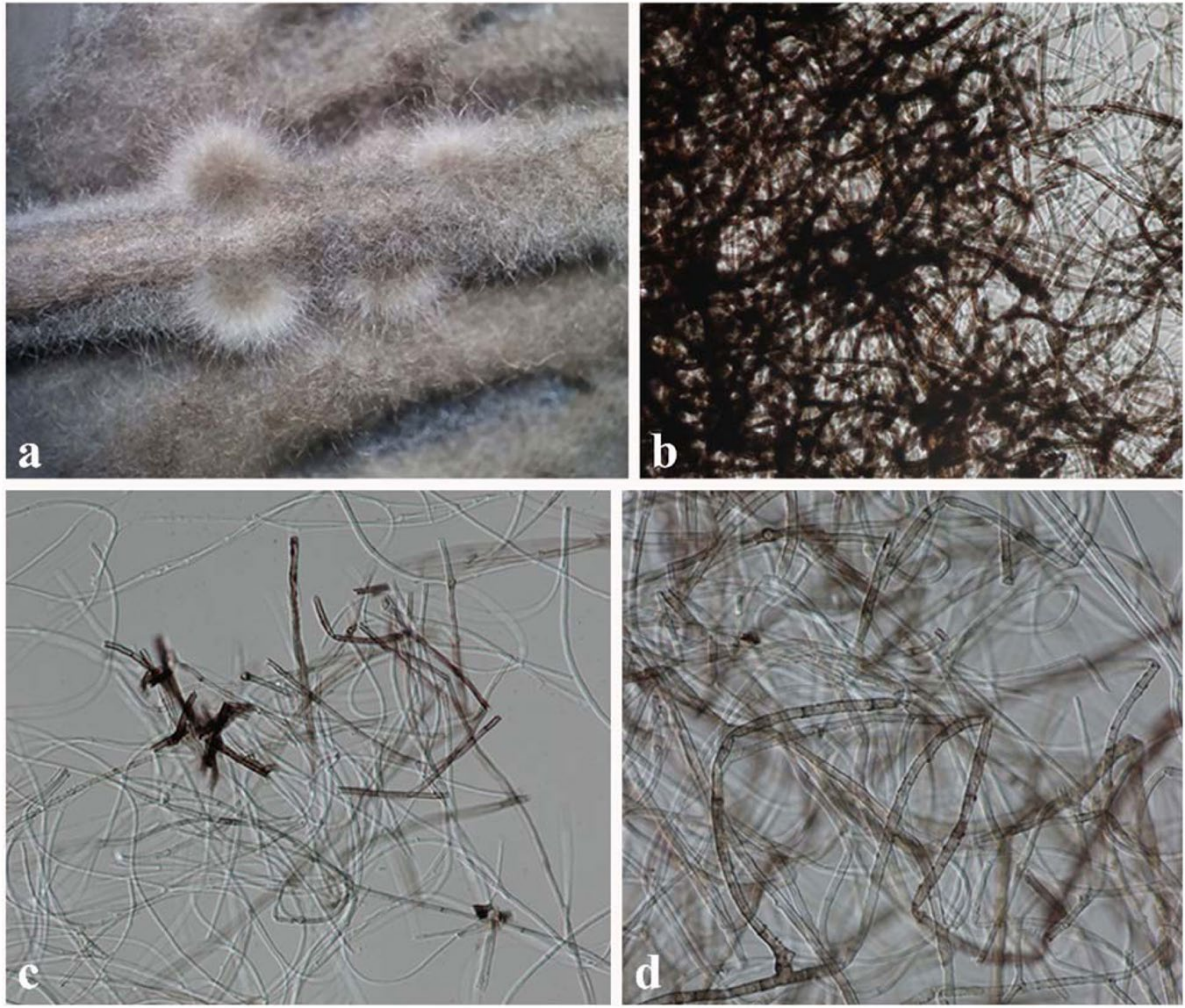
*Lasiodiplodia endophytica* (MFLUCC 18-1121, holotype). (**a)** Conidiomata on bamboo sticks in PDA culture plate. (**b)** Squash mount of conidiomata (**c**,**d)** Fungal mycelia.

Index Fungorum number: IF556218, Faces of Fungi number: FoF 05798 Fig. 6

Etymology – the epithet “endophytica” refers to the endophytic life style of this fungus.

Holotype: MFLU 19-0441

*Endophytic* on fresh leaves of *Magnolia candolii*. Conidiomata not observed on bamboo sticks on PDA, MEA or Water Agar. *Lasiodiplodia endophytica* (S8) clusters with *L*. *iraniensis* and *L*. *thailandica* in a moderately supported clade. It differs from *L*. *iraniensis* by unique fixed alleles in two loci: ITS position 463 (C); *tef1* positions 554 (C), 599 (T), 681 (C), 703 (G) and differs from *L*. *thailandica*: ITS position 463 (C); *tef1* positions 551 (C), 598 (C), 671 (C) 811 (C).

Colonies on PDA reaching 30 mm diameter after 3 days at 20–25 °C, colonies medium sparse, circular, surface slightly rough with edge entire, margin well-defined, cottony to fairly fluffy with sparse aspects, colony from above: grey to black with fluffy appearance; reverse black.

#### Material examined

China, Yunnan Province, Xishuangbanna, fresh leaves of *Magnolia candolii* (Magnoliaceae), 26 April 2017, N. I. de Silva, S8 (Holotype - a dry culture on bamboo sticks - MFLU 19-0441), living cultures, MFLUCC 18-1121, KUMCC 17-0233.

#### Notes

The combined ITS, *tef1* and *tub2* phylogeny showed that *Lasiodiplodia endophytica* (S8) (MFLUCC 18-1121) clusters sister to *Lasiodiplodia iraniensis*. DNA sequence comparisons of ITS and *tef1* among *L*. *endophytica*, *L*. *iraniensis* and *L*. *thailandica* are given in Table 2. Comparison of total length of 477 bases of ITS sequences revealed one base pair difference among three strains and one base deletion in *L*. *endophytica*. Comparison of total length of 290 bases of *tef*1 sequences revealed seven base pair differences among three strains as given in Table 2. This isolate did not sporulate in culture and no conidiomata were seen on the host. Therefore it was not possible to observe conidial characters. The type of *L*. *iraniensis* was isolated from twigs of *Salvadora persica* in Iran^3^. Additionally, *L*. *iraniensis* was recorded from twigs of *Juglans* sp. *Citrus* sp. and *Mangifera indica* in Iran^3^.

**Table 2:** Nucleotide differences between *Lasiodiplodia endophytica* (MFLUCC 18-1121) and ex-type isolates of *L*. *iraniensis* and *L*. *thailandica*.

Locus	ITS		*tef1*- α						
Nucleotide position	420	466	22	25	69	137	138	160	168
*Lasiodiplodia endophytica* MFLUCC 18-1121 (S8)	C	—	C	C	T	C	C	G	C
*Lasiodiplodia iraniensis*	T	T	C	A	A	C	T	C	C
*Lasiodiplodia thailandica*	T	T	A	C	T	T	C	G	T

## Discussion

In this study two new species of *Lasiodiplodia* were identified and described from *Magnolia candolii* in the southern part of Yunnan Province, China. One species (*Lasiodiplodia magnoliae*) was considered to be saprobic, while the other (*L*. *endophytica*) was thought to be endophytic. In addition, two saprobic isolates of *L*. *pseudotheobromae* from dead twigs and an endophytic isolate of the same species from fresh leaves of *Magnolia candolii* were recorded for the first time from China. An endophytic isolate of *Lasiodiplodia thailandica* was also isolated for the first time from fresh leaves of *Magnolia candolii* in China. When Promputtha *et al*.^22^ studied endophytes and Promputtha *et al*.^23^ studied saprobes from leaf litter of *Magnolia liliifera* and *M*. *garretii* respectively in Chiang Mai, Thailand, no *Lasiodiplodia* species were recorded.

Phylogenetic approaches based on DNA sequence data have played a significant role in distinguishing species in *Lasiodiplodia*^3,4,24,25^. Previous studies have used combined ITS and *tef1* regions to clarify the taxonomy and phylogenetic relationships of species in *Lasiodiplodia*^3,6,26^ while others have used combined ITS, *tef1*, *tub2* and *rpb2*^19^. The current phylogenetic analyses with combined ITS, *tef1* and *tub2* sequence data gave good resolution of phylogenetic separations among *Lasiodiplodia* species and provide insights in to taxonomic novelties. Unfortunately, amplification of *tef1* of MFLUCC 18-0951 - C13 and MFLUCC 18-0952 - C17, and *tub2* of MFLUCC 18-0951 - C13 was not successful in this study. Phylogenetic analyses were conducted using DNA sequence data available in GenBank, but unfortunately sequences of *tef1* and *tub2* are not available for some species (see Table S1) and some of the sequences are shorter than expected. These issues of *tef1* and *tub2* might compromise the number of characters in the final alignment and ultimately might affect the final phylogenetic tree construction. We provide phylogenetic analyses for single molecular markers as Supplementary Materials. Phylogenetic trees from ITS and *tub2* did not provided good resolution among *Lasiodiplodia* species. The phylogenetic analysis of ITS gene showed that three newly isolated strains of *Lasiodiplodia pseudotheobromae* and *L*. *thailandica* MFLUCC 18-095 clustered in one group and *L*. *endophytica* MFLUCC 18-1121 clustered separately from that group. *Lasiodiplodia magnoliae* MFLUCC 18-0948 formed a separate clade with *L*. *citricola* IRAN1522C. The phylogenetic analysis of *tub2* did not provide clear separation of newly isolated strains and previously described species. Two *Lasiodiplodia pseudotheobromae* strains, *L*. *thailandica* and *L*. *endophytica* clustered together and *L*. *magnoliae* formed a separate, distantly related lineage. Analysis of *tef1* resulted in a better resolution of many taxa than single ITS and *tub2* gene trees and showed similar phylogenetic relationships as combined ITS, *tef1* and *tub2* analyses. In both *tef1* gene and combined ITS, *tef1* and *tub2* gene phylogenetic analyses, new strains of *Lasiodiplodia pseudotheobromae* formed a clade with other *L*. *pseudotheobromae*, *L*. *endophytica* clustered with *L*. *iraniensis*, *L*. *thailandica* and *L*. *hyalina*. In both *tef1* gene and combined phylogenetic analyses, *Lasiodiplodia magnoliae* reflected similar phylogenetic affiliation with *L*. *chonburiensis*, *L*. *caatinguensis*, *L*. *exigua*, *L*. *pandanicola* and *L*. *mahajangana*. It is also worth noting that the phylogenetic relationships of species within *Lasiodiplodia* recovered herein from combined ITS, *tef1* and *tub2* gene analyses are similar to previously established ones in Dissanayake *et al*.^12^, Dou *et al*.^11^ and Tibpromma *et al*.^10^. These three phylogenetic studies were based on different combinations of molecular markers such as Dissanayake *et al*.^12^ who used combined ITS and *tef1*, Dou *et al*.^11^ used combined ITS, *tef1*, *tub2* and *rpb2* and Tibpromma *et al*.^10^ used ITS, *tef1* and *tub2*. In earlier studies, *Lasiodiplodia* were species distinguished solely on their ITS sequences^10^. In recent studies, taxonomists frequently use highly variable protein coding genes such as *tef1*, *tub2* together with ITS to construct phylogenies especially at species levels^4^. It can be assumed that these combination of molecular markers strengthen the support for them and to separate the existing ones^3,4^.

Conidia of *Lasiodiplodia* species are initially hyaline, aseptate, ellipsoid to ovoid and become pigmented, 1-septate with longitudinal striations^3^. We observed hyaline, aseptate conidia and brown, 1-septate conidia with longitudinal striations in the saprobic isolates of *Lasiodiplodia pseudotheobromae*, but only hyaline conidia were seen in *Lasiodiplodia magnoliae*, the endophytic isolate of *L*. *pseudotheobromae* and *L*. *thailandica*. Previous studies have recorded both hyaline and pigmented conidia in *L*. *pseudotheobromae*^6^ and *L*. *thailandica*^21^. Other *Lasiodiplodia* species have been observed with only hyaline conidia such as *L*. *chonburiensis*^10^, *L*. *sterculiae*^27^ and *L*. *thailandica* in which most conidia were hyaline and only 10% were brown^19^. This character does not seem to be restricted to any particular phylogenetic groups but appears in different *Lasiodiplodia* species. Thus, *L*. *magnoliae* and *L*. *chonburiensis* are closely related and found in one clade. On the other hand, *L*. *sterculiae* and *L*. *thailandica* are distantly related to both *L*. *magnoliae* and *L*. *chonburiensis* and formed widely separate lineages in the phylogenetic tree. *Lasiodiplodia magnoliae* differs from its sister taxa by phylogeny, morphology, host species and locality as described in the notes section that support for the introduction of new saprobic taxa. We were unable to observe conidia of *L*. *endophytica* (S8) in culture even after many attempts on different media. It was considered here that phylogeny based on combined ITS, *tef1* and *tub2* sequence data provides sufficient evidence for the designation of *L*. *endophytica* (S8) as a novel taxon.

*Lasiodiplodia* species exhibit diverse life-styles as endophytes^8,15^, pathogens^3,16^ and saprobes^3,17^. *Lasiodiplodia* species with endophytic life-styles are associated with different asymptomatic plant tissues such as *L*. *avicenniae* from asymptomatic branches of *Avicennia marina* in South Africa, *L*. *bruguierae* from asymptomatic branches of *Bruguiera gymnorrhiza* in South Africa^28^ and *L*. *mahajangana* from healthy branches of *Terminalia catappa* in Madagascar^18^. We isolated three endophytic species; *Lasiodiplodia endophytica*, *L*. *pseudotheobromae* and *L*. *thailandica* from asymptomatic leaves of *Magnolia candolii*. Interestingly, we isolated one endophytic and 2 saprobic isolates of *Lasiodiplodia pseudotheobromae*. This might be possible because endophytes switch their nutritional mode to saprobic when environmental conditions become unfavorable to the host or during host senescence^29^. Thus, de Errasti *et al*.^30^ stated that diatrypaceous endophytic fungi switch to a saprotrophic life-style during host senescence. They explained that this might be ecologically important as they can decay the plant part when it dies^30^. In another scenario, Osorio *et al*.^28^ showed that endophytic *Lasiodiplodia avicenniae* became pathogenic and caused lesions on the branches of *Avicennia marina* after inoculating. It is assumed that some fungi exhibit a continuum of life-styles ranging from biotrophy (or endophytic), through to necrotrophy and ultimately to saprotrophy^29^. Endophytes are a hidden bioresource of fungal diversity that have the potential to produce important bioactive agents^15^. Chen *et al*.^15^ chemically investigated a strain of *Lasiodiplodia* sp. isolated from asymptomatic leaves of the medicinal plant *Acanthus ilicifolius*. They studied β-resorcylic acid derivatives and showed that these compounds showed more potent inhibitory effects against α-glucosidase activity than the clinical α-glucosidase inhibitor acarbose^15^. *Lasiodiplodia* species with pathogenic life-styles are associated with shoot blights, stem cankers, fruit rots, dieback, grapevine trunk diseases and gummosis^3,16,31^ *Lasiodiplodia exigua* from a branch canker of *Retama raetam*^32^, *L*. *mediterranea* from branch canker of *Quercus ilex*^32^, *L*. *plurivora* from V-shaped necrotic lesion of *Prunus salicina*, in Africa^33^ and *L*. *pseudotheobromae* from grapevine trunk disease^16^ are some examples that cause different plant diseases. We did not observe any pathogenic *Lasiodiplodia* species in our study. Saprobic *Lasiodiplodia* species have been recorded such as *Lasiodiplodia iraniensis* on dead twigs of *Salvadora persica*, *L*. *hormozganensis* on *Olea* sp.^3^ and *L*. *theobromae* on dead twigs of *Eucalyptus* sp.^17^. Similarly, we introduced a new saprobic species of *Lasiodiplodia magnoliae* and two isolates of *L*. *pseudotheobromae*.

This study identified *Lasiodiplodia* species in forest plants of *Magnolia candolii* in Yunnan, China. The study has expanded the knowledge of *Lasiodiplodia* species providing two novel species and two new host records. It might be possible to identify new distribution and host associations of *Lasiodiplodia* species from other forest plants in the world. It is important to study endophytic *Lasiodiplodia* species as well as pathogenic and saprobic life-styles as novel endophytes are also yet to be explored.

## Materials and Methods

The study area was a sub-tropical rain forest inside the Xishuangbanna tropical botanical garden in Xishuangbanna at 21°55′N, 101°15′E, Yunnan province, China. Elevation ranges from 709–869 m and mean temperature and precipitation are 21.0 °C and 1532 mm respectively. The wet season is from May to October while the dry season is from November to April^34,35^.

### Isolation of fungal endophytes

Fresh leaves of *Magnolia candolii* were collected randomly on 26^th^ of April 2017. The leaves were kept at 4 °C in sterile polyethylene bags until they were processed in the laboratory. Isolation of endophytes was done according to the methods described by Promputtha *et al*.^36^ with modifications. First leaves were washed using tap water and cut in to small pieces of leaves (5 × 5 mm^2^) and soaked in distilled water for 1 minute and then surfaced sterilized by dipping in 70% alcohol followed by 2% NaOCl for 30 s and finally washed with sterile distilled water for 30 s, dried and plated on Potato Dextrose Agar (PDA). Fungi were isolated into pure culture and grouped according to their culture morphology.

### Isolation of fungal saprobes

Fungi were isolated from dead twigs attached to the host. Specimens were taken to the laboratory in Ziplock plastic bags and observed with a JNOEC JSZ4 stereomicroscope. Micro-morphological characters were examined with an OLYMPUS SZ61 compound microscope and images recorded with a Canon EOS 600D digital camera mounted on a Nikon ECLIPSE 80i compound microscope. All microscopic measurements were made with Tarosoft (R) image framework v. 0.9.0.7 and images for publication were processed with Adobe Photoshop CS3 extended version. Pure cultures of the fungus were prepared by single spore isolation^37^. Germinating conidia were transferred aseptically to potato dextrose agar (PDA). Growth rate and colony characteristics were determined from cultures grown on PDA at room temperature (25 °C) for one week.

The specimens cited in this paper are maintained at the Mae Fah Luang University Herbarium (MFLU), Chiang Rai, Thailand and Kunming Institute of Botany herbarium (HKAS), Kunming, China. Cultures were deposited at Kunming Institute of Botany Culture Collection (KUMCC). Faces of Fungi numbers and Index Fungorum numbers were registered as described in Jayasiri *et al*.^38^ and Index Fungorum (2019)^39^.

### DNA extraction, PCR amplification and phylogenetic analysis

Mycelium was grown on PDA for one week at 25 °C in normal light in the laboratory. Genomic DNA was extracted from the mycelium using a Biospin fungus genomic DNA kit (BioFlux®, P.R. China) following the manufacturer’s protocol. DNA was kept at 4 °C for DNA amplification and maintained at −20 °C for long term storage.

The internal transcribed spacer (ITS) was amplified with primer pair ITS4 and ITS5^40^ as described in Alves *et al*.^41^. Part of the translation elongation factor (*tef1*) was amplified with primer pair EF1-728F and EF1-986 Carbone and Kohn^42^ and EF1-688F and EF1-1251R Alves *et al*.^6^. The partial β-tubulin (*tub2*) was amplified with primer pair Bt2a and Bt2b^43^. The expected sequence lengths are approximately 500 bp, 300–400 bp, 400 bp for ITS, *tef1* and *tub2* respectively. Quality of PCR products was checked on 1% agarose electrophoresis gels stained with ethidium bromide. The amplified PCR fragments were sequenced by Sangon Biotech (Shanghai) Co., Ltd, P.R. China.

Newly generated nucleotide sequences were deposited in GenBank (Table S1 in Supplementary material). Sequences of the individual loci of ITS, *tef1* and *tub2* were aligned with MAFFT v. 7 online version^44^ using default settings. BioEdit v. 7.0.5.2^45^ was used to refine the alignments manually where necessary and to exclude incomplete portions at the ends of the sequences before the analyses.

Maximum likelihood analysis was performed with RAxML GUI v. 1.3^46^ and maximum parsimony analysis was done with PAUP (Phylogenetic Analysis Using Parsimony) v. 4.0b10^47^. Evolutionary models for phylogenetic analyses were selected independently for each locus using MrModeltest v. 3.7^48^ under the Akaike Information Criterion (AIC). GTR + I + G model of nucleotide substitution was selected for the maximum likelihood (ML) analysis. Parameters for maximum likelihood were set to rapid bootstrapping and the analysis carried out using 1000 replicates. Maximum parsimony was run with the heuristic search option, random taxon addition, tree bisection-reconnection (TBR) for the branch swapping algorithm and 1000 random sequence additions, with maxtrees set at 1000. Gaps were treated as missing data. Tree Length [TL], Consistency Index [CI], Retention Index [RI], Relative Consistency Index [RC] and Homoplasy Index [HI] were calculated for the most parsimonious tree. Phylograms were visualized with FigTree v1.4.0 Rambaut^49^ and annotated in Microsoft Power Point (2010). The alignment and tree files were submitted to TreeBASE with reviewer’s link (http://purl.org/phylo/treebase/phylows/study/TB2: S23955).

## Supplementary information

GenBank accession number and single gene phylogenetic trees
