# Supplementary material for: Phylogeny and morphology of Lasiodiplodia species associated with Magnolia forest plants
Source: Sci Rep. 2019 Oct 4;9:14355. doi: 10.1038/s41598-019-50804-x (PMC6778208; doi:10.1038/s41598-019-50804-x)
Supplement: Supplementary file 1 — GenBank accession number and single gene phylogenetic trees [file 41598_2019_50804_MOESM1_ESM.docx]

**Phylogeny and morphology of *Lasiodiplodia* species associated with *Magnolia* forest plants**

**Nimali I. de Silva^1,2,3,4,5^, Alan J. L. Phillips^6^, Jian-Kui Liu^7^, Saisamorn Lumyong^1,2,8,*^ & Kevin D. Hyde^1,3,4,5^**

^1^Department of Biology, Faculty of Science, Chiang Mai University, Chiang Mai, 50200 Thailand

^2^Center of Excellence in Microbial Diversity and Sustainable Utilization, Faculty of Science, Chiang Mai University, Chiang Mai, 50200 Thailand

^3^Key Laboratory for Plant Biodiversity and Biogeography of East Asia (KLPB), Kunming Institute of Botany, Chinese Academy of Science, Kunming 650201, P.R. China

^4^Center of Excellence in Fungal Research, Mae Fah Luang University, Chiang Rai, 57100, Thailand

^5^World Agro Forestry Centre, East and Central Asia, 132 Lanhei Road, Kunming 650201, P.R. China

^6^Universidade de Lisboa, Faculdade de Ciências, Biosystems and Integrative Sciences Institute (BioISI), Campo Grande, 1749- 016 Lisbon, Portugal

^7^Center for Bioinformatics, School of Life Science and Technology, University of Electronic Science and Technology of China, Chengdu 611731, P.R. China

^8^Academy of Science, the Royal Society of Thailand, Bangkok, 10300, Thailand

*****corresponding author scboi009@gmail.com

Supplementary materials

**Table S1**. GenBank accession numbers and culture accession numbers of isolates included in this study. The newly generated sequence is shown in black bold.

| Species | Isolate | Genbank accession number | | |
| --- | --- | --- | --- | --- |
|  |  | ITS | *tef1* | *tub2* |
| *Diplodia mutila* | CMW 7060 | AY236955 | AY236904 | AY236933 |
| *L. americana* ^T^ | CERC 1961 | KP217059 | KP217067 | - |
| *L. avicenniae* ^T^ | CBS 139670/CMW41467 | KP860835 | KP860680 | KP860758 |
| *L. brasiliense* ^T^ | CMM 4015 | JX464063 | JX464049 | - |
| *L. bruguierae* ^T^ | CBS 139669 | KP860832 | KP860677 | KP860755 |
| *L. caatinguensis* ^T^ | CMM 1325 | KT154760 | KT008006 | KT154767 |
| *L. chinensis* ^T^ | CGMCC 3.18061 | KX499889 | KX499927 | KX500002 |
| *L. chonburiensis* ^T^ | MFLUCC 16-0376 | MH275066 | MH412773 | MH412742 |
| *L. cinnamomi* ^T^ | CFCC 51997 | MG866028 | MH236799 | MH236797 |
| *L. citricola* ^T^ | CBS 124707/ IRAN1522C | GU945354 | GU945340 | KU887505 |
| *L. crassispora* ^T^ | CBS 118741/WAC12533 | DQ103550 | EU673303 | KU887506 |
| *L. euphorbiicola* ^T^ | CMM 3609 | KF234543 | KF226689 | KF254926 |
| *L. euphorbiicola* | CMM 2275 | KC484843 | KC481567 | - |
| ***L. endophytica* ^T^** | **MFLUCC 18-1121** | **MK501838** | **MK584572** | **MK550606** |
| *L. exigua* ^T^ | CBS 137785 / BL104 | KJ638317 | KJ638336 | KU887509 |
| *L. gilanensis* ^T^ | CBS 124704 /IRAN1523C | GU945351 | GU945342 | KU887511 |
| *L. gonubiensis* ^T^ | CBS 115812 /CMW14077 | AY639595 | DQ103566 | DQ458860 |
| *L. gravistriata* ^T^ | CMM 4564 | KT250949 | KT250950 | - |
| *L. hormozganensis* ^T^ | CBS 124709 / IRAN1500C | GU945355 | GU945343 | KU887515 |
| *L. hyaline* ^T^ | CGMCC 3.17975 | KX499879 | KX499917 | KX499992 |
| *L. iraniensis* ^T^ | CBS 124710/ IRAN921 | GU945346 | GU945334 | KU887516 |
| *L. iraniensis* | CMM 3610 | KF234544 | KF226690 | KF254927 |
| *L. laeliocattleyae* ^T^ | CBS 167.28 | KU507487 | KU507454 | - |
| *L. laeliocattleyae* ^T^ | CBS 130992/BOT10 | JN814397 | JN814424 | KU887508 |
| *L. lignicola* ^T^ | CBS 134112 /MFLUCC 11-0435 | JX646797 | KU887003 | JX646845 |
| *L. macrospora* ^T^ | CMM 3833 | KF234557 | KF226718 | KF254941 |
| ***L. magnoliae* ^T^** | **MFLUCC18-0948/KUMCC17-0198** | **MK499387** | **MK568537** | **MK521587** |
| *L. mahajangana* ^T^ | CBS 124927 / CMW27801 | FJ900595 | FJ900641 | FJ900630 |
| *L. margaritacea* ^T^ | CBS 122519 | EU144050 | EU144065 | KU887520 |
| *L. mediterranea* ^T^ | CBS 137783 / BL1 | KJ638312 | KJ638331 | KU887521 |
| *L. missouriana* ^T^ | CBS 128311 /UCD2193MO | HQ288225 | HQ288267 | HQ288304 |
| *L. pandanicola* ^T^ | MFLUCC 16-0265 | MH275068 | MH412774 | MH412744 |
| *L. parva* ^T^ | CBS 456.78 | EF622083 | EF622063 | KU887523 |
| *L. plurivora* ^T^ | CBS 120832 / STE-U 5803 | EF445362 | EF445395 | KP872421 |
| *L. pontae* ^T^ | CMM 1277 | KT151794 | KT151791 | KT151797 |
| *L. pseudotheobromae* ^T^ | CBS 116459 | EF622077 | EF622057 | EU673111 |
| *L. pseudotheobromae* | CBS 304.79 | EF622079 | EF622061 | - |
| *L. pseudotheobromae* | CBS 374.54 | EF622080 | EF622059 | - |
| *L. pseudotheobromae* | CBS 116460 | EF622078 | EF622058 | **-** |
| *L. pseudotheobromae* | IBL 241 | KT247479 | KT247483 | KT247489 |
| *L. pseudotheobromae* | CJA 36 | GU973875 | GU973867 | **-** |
| ***L. pseudotheobromae -* NI173** | **MFLUCC 18-1120** | **MK496933** | **MK521585** | **MK524719** |
| ***L. pseudotheobromae -* NI173A** | **MFLUCC 18-0950** | **MK501818** | **MK521586** | **MK550605** |
| ***L. pseudotheobromae -*  C13** | **MFLUCC 18-0951** | **MK501819** | **-** | **-** |
| *L. pyriformis* ^T^ | CBS 121770 | EU101307 | EU101352 | KU887527 |
| *L. rubropurpurea* ^T^ | CBS 118740 / WAC12535 | DQ103553 | EU673304 | EU673136 |
| *L. sterculiae* ^T^ | CBS 342.78 | KX464140 | KX464634 | KX464908 |
| *L. subglobosa* ^T^ | CMM 3872 | KF234558 | KF226721 | KF254942 |
| *L. thailandica* ^T^ | CBS 138760/ CGMCC 3.18384 | KY767663 | KY751304 | KY751301 |
| ***L. thailandica*** | **MFLUCC18-0952** | **MK501839** | **-** | **MK524720** |
| *L. theobromae* ^T^ | CBS 164.96 | AY640255 | AY640258 | KU887532 |
| *L. venezueiensis* ^T^ | CBS 118739/ WAC12539 | DQ103547 | EU673305 | KU887533 |
| *L. viticola* ^T^ | CBS 128313 / UCD2604MO | HQ288228 | HQ288270 | HQ288307 |
| *L. vitis* ^T^ | CBS 124060 | KX464148 | KX464642 | KX464917 |

^T^ Ex-type strains


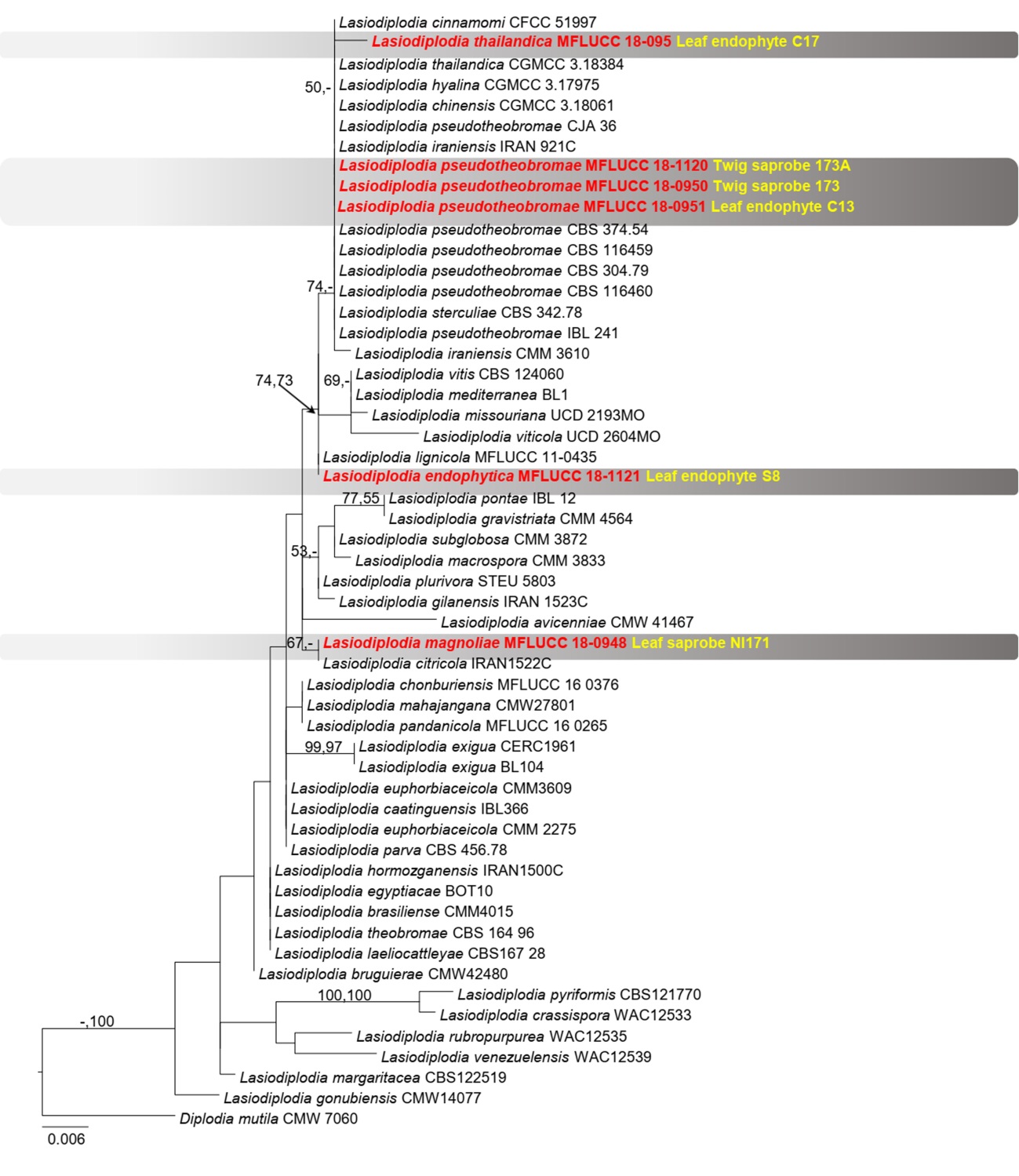


**Figure1.** Maximum likelihood tree resulting from ITS data alignment. Bootstrap values for maximum likelihood (ML, first set) greater than 50, and maximum parsimony (MP, second set) greater than 50 are indicated at the nodes. The tree is rooted with *Diplodia mutila* (CMW 7060).


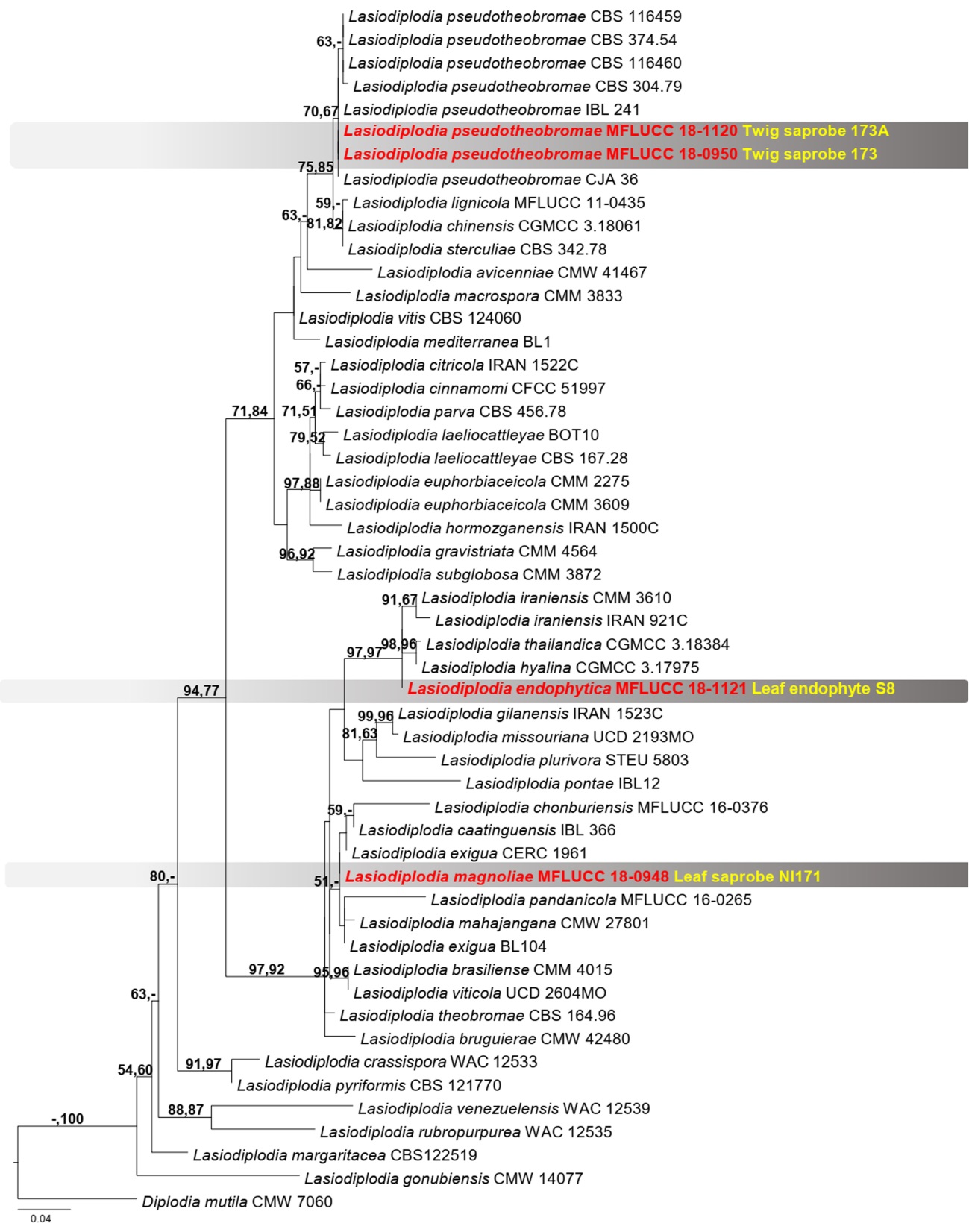


**Figure 2.** Maximum likelihood tree resulting from *tef1* data alignment. Bootstrap values for maximum likelihood (ML, first set) greater than 50, and maximum parsimony (MP, second set) greater than 50 are indicated at the nodes. The tree is rooted with *Diplodia mutila* (CMW 7060).


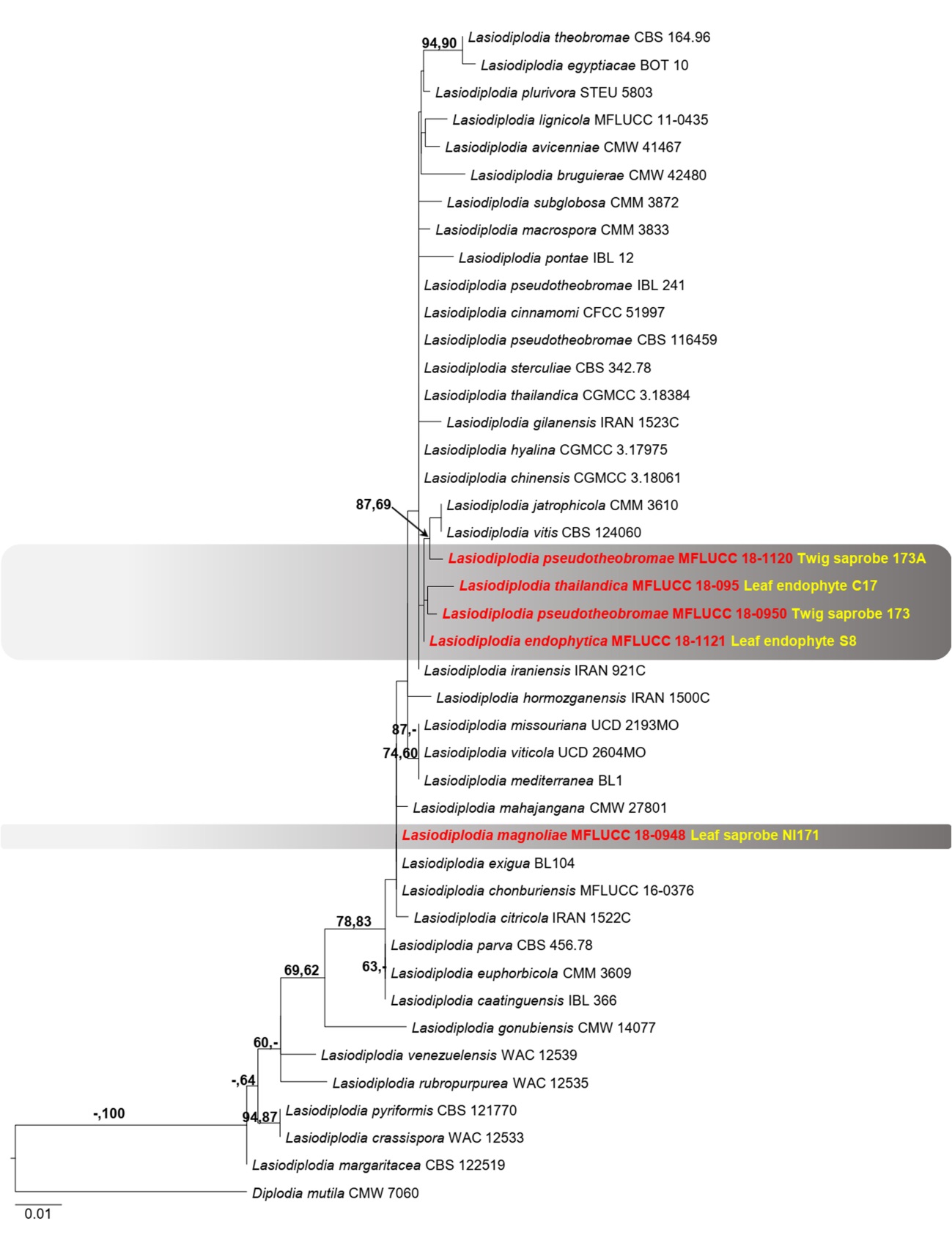


**Figure 3.** Maximum likelihood tree resulting from partial β-tubulin (*tub2*) data alignment. Bootstrap values for maximum likelihood (ML, first set) greater than 50, and maximum parsimony (MP, second set) greater than 50 are indicated at the nodes. The tree is rooted with *Diplodia mutila* (CMW 7060).
